# The support of medication reviews in hospitalised patients using a clinical decision support system

**DOI:** 10.1186/s40064-016-2376-1

**Published:** 2016-06-24

**Authors:** Hugo A. J. M. de Wit, Kim P. G. M. Hurkens, Carlota Mestres Gonzalvo, Machiel Smid, Walther Sipers, Bjorn Winkens, Wubbo J. Mulder, Rob Janknegt, Frans R. Verhey, Paul-Hugo M. van der Kuy, Jos M. G. A. Schols

**Affiliations:** Department of Clinical Pharmacy and Toxicology, Zuyderland Medical Centre, Henri Dunantstraat 5, 6419 PC Heerlen, The Netherlands; Department of Internal Medicine, Section of Geriatric Medicine, Zuyderland Medical Centre, Sittard-Geleen, The Netherlands; Department of Clinical Pharmacy and Toxicology, Zuyderland Medical Centre, Sittard-Geleen, The Netherlands; Department of Methodology and Statistics, CAPHRI-School for Public Health and Primary Care, Maastricht University, Maastricht, The Netherlands; Department of Internal Medicine, Maastricht University Medical Centre, Maastricht, The Netherlands; Department of Psychiatry and Neuropsychology, Alzheimer Centrum Limburg/School for Mental Health and Neurosciences, Maastricht University, Maastricht, The Netherlands; Department of General Practice and Department of Health Services Research, CAPHRI-School for Public Health and Primary Care, Maastricht University, Maastricht, The Netherlands

**Keywords:** Clinical decision support systems, Medication errors, Geriatrics

## Abstract

**Objectives:**

First, to estimate the added value of a clinical decision support system (CDSS) in the performance of medication reviews in hospitalised elderly. Second, to identify the limitations of the current CDSS by analysing generated drug-related problems (DRPs).

**Methods:**

Medication reviews were performed in patients admitted to the geriatric ward of the Zuyderland medical centre. Additionally, electronically available patient information was introduced into a CDSS. The DRP notifications generated by the CDSS were compared with those found in the medication review. The DRP notifications were analysed to learn how to improve the CDSS.

**Results:**

A total of 223 DRP strategies were identified during the medication reviews. The CDSS generated 70 clinically relevant DRP notifications. Of these DRP notifications, 63 % (44) were also found during the medication reviews. The CDSS generated 10 % (26) new DRP notifications and conveyed 28 % (70) of all 249 clinically relevant DRPs that were found. Classification of the CDSS generated DRP notifications related to ‘medication error type’ revealed that ‘contraindications/interactions/side effects’ and ‘indication without medication’ were the main categories not identified during the manual medication review. The error types ‘medication without indication’, ‘double medication’, and ‘wrong medication’ were mostly not identified by the CDSS.

**Conclusions:**

The CDSS used in this study is not yet sufficiently advanced to replace the manual medication review, though it does add value to the manual medication review. The strengths and weaknesses of the current CDSS can be determined according to the medication error types.

## Background

Frailty in the aging patient is a state of vulnerability in which health status can suddenly decrease as a result of relatively small health events (Clegg et al. 2013). Ultimately frailty may lead to considerable disability. Frail elderly often have multiple chronic conditions which are associated with the use of many drugs. Polypharmacy is often defined as the use of more than five drugs per patient. This is an arbitrarily chosen cut-off point and varies between studies. Alternatively, polypharmacy can be defined as the use of a higher number of drugs than clinically indicated (Maher et al. 2014; Hajjar et al. 2007). Both polypharmacy and frailty are independently associated with morbidity intensity suggesting a direct effect of polypharmacy on a patient’s frailty status. The influence of polypharmacy on frailty might be explained by decreased compliance, more adverse drug reactions (ADR), and drug interactions. Excessive polypharmacy, which is the use of more than ten drugs, is an independent risk factor for mortality (Herr et al. 2015).

A drug-related problem (DRP) is “an event or a circumstance involving drug therapy that actually or potentially interferes with health care outcome” (Silva et al. 2015). DRPs are associated with (frail) elderly as a consequence of polypharmacy, complex dosing regimens as well as alterations in pharmacokinetics and pharmacodynamics. DRPs are also associated with cognitive and functional status (Silva et al. 2015).

Leendertse et al. showed that polypharmacy is an independent risk factor for hospitalisation. Of the unplanned hospital admissions 5.6 % were medication-related. Of these almost half were probably preventable (Leendertse et al. 2008). Paradoxically, polypharmacy has been shown to increase during hospitalisation of elderly (Nobili et al. 2011).

### Medication review

A medication review is defined as a structured evaluation of a patient’s medication by a physician and a pharmacist, taking into account medical history and laboratory values. The medication review aims to reach agreement about drug therapy in order to optimise the impact of medication while minimising the number of medication-related problems. When the patient’s input into the medication review is included with all available patient’s information, the review is defined as a clinical medication review. Without the patient’s input, the review is defined as a treatment review. This is often the case in hospitalised patients (Blenkinsopp et al. 2012; van Dijk et al. 2009). It has been suggested that there should be an intermediate definition for hospitalised patients, since patients are followed up daily by nursing staff and physicians, while recognising that dementing or very ill patients cannot give comments on their medication (van Dijk et al. 2009). In a systematic review by Christensen et al. no conclusive benefits on future hospitalisations or mortality in hospitalised patients are shown when performing medication reviews. However, performing medication reviews has shown to reduce the number of emergency department contacts (Christensen and Lundh 2013). Furthermore, it has been shown that regular revision of medication reduces DRPs and improves medication appropriateness (Alldred et al. 2013). The inconsistency of proven benefits of medication reviews might be caused by implementation problems. These problems involve the time efficiency of performing medication reviews as well as the structure and information used in a medication review. The output consistency of the health care professionals performing the medication reviews can also be a factor (de Wit et al. 2014b; Hurkens et al. 2013; Mestres Gonzalvo et al. 2015). These problems might be (partially) resolved by automation of the medication review (de Wit et al. 2013).

### Clinical decision support systems (CDSS)

The Dutch Healthcare Inspectorate (IGZ) requires physicians to prescribe exclusively using computerised physician order entry (CPOE) systems with an integrated drug safety alert system.

In the Netherlands, a nationwide drug database is maintained by the Royal Dutch Association for the Advancement of Pharmacy (KNMP) that generates drug safety alerts to ensure medication surveillance which include dosage appropriateness, double medication, drug–drug interactions, and drug contraindications (Richtlijn elektronisch voorschrijven 2013). The pharmacist responsible for the CPOE can regulate the drug safety alerts to a degree (Eppenga et al. 2012). These alerts result from relatively uncomplicated algorithms that form a basic clinical decision support system (CDSS) (Kuperman et al. 2007). The use of a CDSS with advanced algorithms that combine medication and laboratory values is becoming common practice in the Netherlands, and the use of advanced algorithms that incorporate guidelines and drug–disease interactions that initiate treatment to prevent adverse drug events is up-coming (Rommers et al. 2011; O’Sullivan et al. 2014; Meulendijk et al. 2015). Unlike the basic algorithms that are routinely integrated into CPOEs, the advanced algorithms are mostly not integrated into a CPOE. Integration of a CDSS into a CPOE results in the presentation of alerts when a physician is prescribing medication. In this way the physician can adjust the prescription during the prescription process if necessary, instead of adjusting the prescription afterwards, such as when it is initiated by an alert from a stand-alone CDSS (de Wit et al. 2015; Eppenga et al. 2012; Tawadrous et al. 2011). Patient outcome benefits of using advanced CDSSs remain limited, but several studies do report positive results related to prevention of adverse drug events (Rommers et al. 2011; Tawadrous et al. 2011; Bright et al. 2012; Jaspers et al. 2011). Bright et al. (2012) have shown in a systematic review that CDSSs do influence ‘health care process measures’ by improving the initiated treatment or preventive care in terms of: pharmacotherapy, laboratory test ordering, and chronic disease management.

In a recent study, the use of an advanced CDSS to support medication reviews in older hospitalised patients has shown to improve the appropriateness and accuracy of medication regimens (O’Sullivan et al. 2014).

The department of clinical pharmacy and toxicology of the Zuyderland medical centre has developed an advanced CDSS based on our experience with a simpler access based CDSS (de Wit et al. 2015). The currently developed advanced CDSS consists of algorithms that alert for the inappropriate combination of medication and laboratory values, for lack of guideline implementation, and gives suggestions to prevent adverse drug events as a result of drug–disease interactions. The development phases of the CDSS have been described in more detail previously (de Wit et al. 2013). The content development team consisted of several hospital pharmacists and internists, a neuropsychiatrist, and a nursing home physician. In the Netherlands, nursing home medicine is an officially recognised medical discipline for physicians attending nursing homes (Schols et al. 2004). The content was based on national guidelines, protocols and relevant studies. The CDSS consists of 469 clinical rules aiming to have a high sensitivity and specificity. This standalone CDSS has been developed to support medication reviews in synergy with the already integrated CPOE drug safety alerts that consider dosage appropriateness, double medication, drug–drug interactions, and drug contraindications.

The aim of this study was to estimate the value of this CDSS regarding the performance of medication reviews in hospitalised elderly by comparing DRPs taken from a multidisciplinary treatment review to the DRP notifications prompted by the CDSS. Furthermore, we aimed to identify the limitations of the current CDSS by analysing the CDSS generated DRP notifications.

## Methods

From November 2012 to December 2013 medication reviews were performed in clinical patients admitted to the geriatric ward of the Zuyderland medical centre, located in Sittard-Geleen. The medication reviews were performed during the weekly ‘gerontopharmacology meeting’. During every gerontopharmacology meeting one patient was discussed for 0.5–1 h. The patients were selected by the geriatrician and communicated to all the participants before the meeting. At least one geriatrician (leading practitioner and chairman of the meeting), one resident and one hospital pharmacist (in training) attended the meeting. In most cases, more clinicians attended, including geriatricians, residents, nurse practitioners and medical students. The meeting was also intended to provide educational value. The leading practitioner and chairman of the meeting was either a geriatrician or an internist, with a subspecialty in old age medicine. Both are geriatric experts, and have expertise in medical education. The attending hospital pharmacist had extensive experience in performing structured medication reviews, as necessary for this study. Apart from these experts, up to 5 postgraduate physician residents and 5 medical students attended the meeting.

The structure of the review was based on the method used in the PHARM-study and was followed during the medication reviews (Leendertse et al. 2011). This structure was: (1) Matching the prescribed medication with the known indications, (2) Matching indications with prescribed medications, (3) Relating the laboratory values to the prescribed medication, (4) A general discussion whether there were other suggestions based on the patients’ or nurses input. Although the pharmacist did not perform patients’ interviews, the geriatrician and residents had daily contact with the reviewed patients, and any relevant information was then presented in the meeting. The indirect input of patients’ comments causes these medication reviews to supersede the requirements of a standard “treatment review”, but does not meet the requirements of a “clinical medication review”. For this study, we will continue to address them as medication reviews.

The hospital pharmacist prepared the medication review by documenting the DRP suggestions resulting from the manual check of medications, laboratory values, and other relevant clinical patient information. These suggestions were discussed during the medication review by the attendants. If they were accepted by the attendants as a DRP, it was included as a strategy to improve the patient’s therapy. The accepted ‘DRP strategies’ were documented by the hospital pharmacist. A week after the medication review the hospital pharmacist documented which DRPs had indeed been executed. In this study, we will discuss the DRPs using the following terms: “DRP suggestions” (when DRPs were identified during the pharmacist’s preparation), “DRP remarks” (newly introduced DRPs during the medication review), “DRP strategies” (when DRPs were accepted as therapy strategies for the patient during the medication review), “DRP notifications” (the DRPs found by the CDSS), and “DRPs executed” (the carried out therapy strategies based on the DRP strategies).

In addition, prior to the gerontopharmacology meeting the hospital pharmacist extracted all electronic available patient information: laboratory values, medication, and documented indications and contraindications. The extracted electronic patient information was introduced into the CDSS in April 2015, therefore it was not possible that the medication reviews identified DRPs were influenced by the CDSS generated DRPs. The CDSS is designed to exclusively alert for relevant DRPs (DRP notifications). This is done by including ‘triggers’ that allow assessing whether the predefined DRPs are relevant or non-relevant. For example, there is an algorithm alerting for the prescription of a gastrointestinal prophylaxis in a patient at risk. However, for some patients a prophylaxis will already be prescribed and this notification is therefore assessed as a non-relevant notification by the CDSS. The DRP notifications from the CDSS were independently checked for relevancy and classified for type of medication error by hospital pharmacist HW and geriatrician KM. In case of disagreement, consensus was obtained by discussing on a case by case basis.

The local Medical Research & Ethics Committee (MREC) determined this study to be non-accessory for the Dutch Medical Research in Human Subjects Act (non-WMO).

Numerical variables were presented by mean (SD; range, i.e. minimum–maximum value) and categorical ones by number (%). The descriptive statistics were computed using Microsoft Excel 2010.

## Results

We held 33 documented gerontopharmacology meetings during which 33 medication reviews were performed, all on different patients. The mean age of the reviewed patients was 83 years (SD 8.0; range 69–97). Gender was distributed almost equally with 55 % (n = 18) of the patients being male. The mean amount of prescribed drugs per patient was 15.2 (SD 4.0; range 8–27 medicines).

### Drug-related problems

**Fig. 1:**
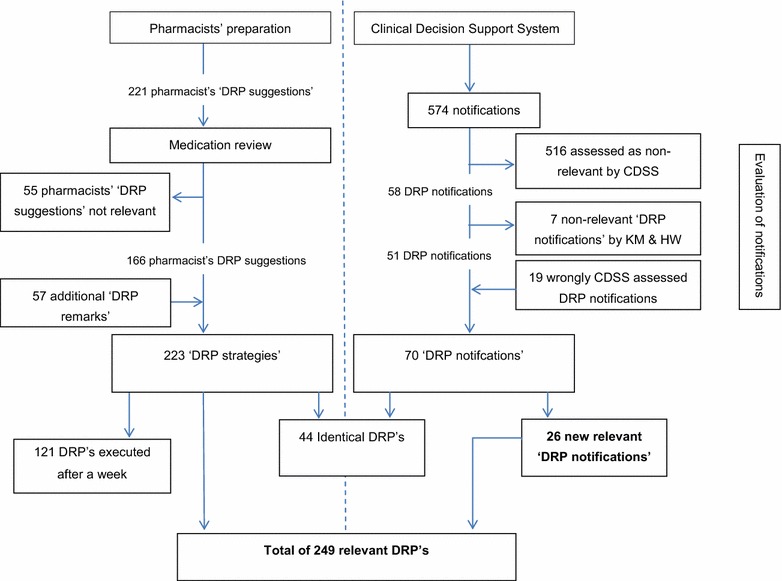
Flowchart of DRPs from 33 medication reviews

The pharmacist who prepared the gerontopharmacology meeting had a total of 221 DRP suggestions based on the 33 medication reviews. Of these 221 DRP suggestions, 166 DRP suggestions were accepted as DRP strategies, while 55 DRP suggestions were rejected and an additional 57 DRP remarks were introduced during the meeting and accepted as DRP strategies. Thus, a total of 223 DRP strategies were accepted with a mean of 7.0 (SD 2.2; range 2–11) per patient (see Fig. 1).

The CDSS generated 574 DRP notifications. Of these notifications the CDSS determined 516 to be non-relevant notifications. The remaining 58 DRP notifications were determined as relevant DRP notifications by the CDSS. These 58 CDSS DRP notifications were checked by HW and KM, 51 of which were considered clinically relevant for the patient. In addition, HW and KM confirmed 19 notifications that were assessed as non-relevant by the CDSS to be clinically relevant DRP notifications. In total 70 DRP notifications issued by the CDSS were relevant (see Fig. 1).

Of the confirmed relevant CDSS DRP notifications, 63 % (44) DRP notifications were also noted and accepted as DRP strategies in the medication reviews. Twenty-six DRP notifications from the CDSS had not been identified during the medication review but were assessed as clinically relevant by HW and KM. Table 1 shows the CDSS DRP notifications that went unnoticed in the medication reviews.

**Table 1:** DRP notifications not identified in the medication review

Potassium level—drugs inducing hypokalemia
Use of aspirin, dipyridamol, clopidogrel, prasugrel without a statin
Calcium channel blockers with chronic constipation
Atypical antipsychotics combined with oral blood glucose lowering drugs or insulin
Atypical antipsychotics combined with Alzheimer medication
Nitrate without aspirin or clopidogrel, prasugrel, ticagrelor
Metformin with unknown vitamin B12 level
Renal failure with amoxicillin (oral)
Atypical antipsychotics combined with antihypertensive medication
Nitrate without a beta blocker
Nortriptyline usage in elderly patients
Tricyclic antidepressants with constipation
Paracetamol in elderly patients in combination with risk factors
Gastric protection without prophylaxis
Gastric protection, prophylaxis dosage not high enough
Classical antipsychotics with anticholinergic effects—start alpha blockers may be related to this effect
Tricyclic antidepressants with cardiac conductive abnormalities
Use of rheumatoid arthritis drugs without a statin
Bisphosphonates, calcium and vitamin D supplementation: suggest prescribing calcium
Renal failure with metoclopramide

These 44 DRP notifications covered 20 % of the DRP strategies identified in the medication review. The CDSS generated an additional 26 newly confirmed DRP strategies (see Fig. 1). The 223 DRP strategies from the medication reviews combined with the additional 26 confirmed relevant DRP notifications from the CDSS, add up to a total of 249 DRP strategies. The CDSS generated 28.1 % (70) of all 249 DRPs strategies of which 10 % (26) were new notifications.

The 223 DRP strategies determined in the medication reviews were followed up for implementation a week after the medication review. Fifty-five percent (121) of these DRP strategies were executed a week after the medication review, which is a mean 3.8 (SD 2.3; range 0–9) DRPs executed per patient.

### Classification of drug-related problems

All DRPs were categorised according to a seven-fold ‘medication error type’ classification (see Table 2). Table 2 shows that two medication error types (a) ‘indication without medication’ and (b) ‘medication without indication’ made up more than half of the existing medication errors. The 26 relevant DRP notifications not recognised during the medication reviews were mainly medication errors type (c) ‘contraindications/interactions/side effects’ and error type (a) ‘indication without medication’. The error types (b) ‘medication without indication’, (e) ‘double medication’, and (f) ‘wrong medication’ were least likely to be identified by the CDSS.

**Table 2:** Classification of medication error types

	Type of error	All remarks = 308	Notifications CDSS and review = 249	CDSS total = 70	Review total = 223	CDSS new notifications N = 26	No notifications in CDSS as % of review remarks (n = 179 vs. n = 223)
a	Indication without medication	94 (30.5 %)	76 (30.5 %)	29 (41 %)	68 (30.5 %)	8 (31 %)	47 (69.1 %)
b	Medication without indication	78 (25.3 %)	66 (26.5 %)	2 (3 %)	66 (29.6 %)	0	64 (97.0 %)
c	Contraindications/interactions/side effects	32 (10.4 %)	25 (10.0 %)	20 (29 %)	11 (4.9 %)	14 (54 %)	5 (45.5 %)
d	Dosage problems	42 (13.6 %)	35 (14.1 %)	14 (20 %)	31 (13.9 %)	4 (15 %)	21 (67.7 %)
e	Double medication	10 (3.2 %)	7 (2.8 %)	0	7 (3.1 %)	0	7 (100 %)
f	Wrong medication	26 (8.4 %)	22 (8.8 %)	1 (1 %)	22 (9.9 %)	0	21 (95.5 %)
g	Therapeutic drug monitoring	26 (8.4 %)	18 (7.2 %)	4 (6 %)	18 (8.1 %)	0	14 (77.8 %)

The columns shows type of medication errors identified during the medication review and by our CDSS independently of the medication review. The last column shows the percentage of medication error types our CDSS did not identify

### CDSS efficiency

The efficiency of the CDSS was determined by calculating the sensitivity and specificity of the CDSS using the confirmed correctness of CDSS notifications. Fifty-one of the 58 DRP notifications were found true positive, giving a sensitivity of 72.9 %. Of the 516 DRPs assessed as non-relevant notifications by the CDSS, 19 notifications were confirmed relevant by KH and HW giving 497 DRP true negative notifications resulting in a specificity of 98.6 %. In Table 3 the sensitivity and specificity are shown.

**Table 3:** Sensitivity = A/A + C × 100 % = 72.9 %, specificity = D/D + B × 100 = 98.6 %

	Confirmed relevant DRPs	Confirmed irrelevant DRPs	
Relevant DRP notifications	51 (A)	7 (B)	58 (A + B)
Non-relevant DRP notifications	19 (C)	497 (D)	516 (C + D)
	70 (A + C)	504 (B + D)	N = 574

The confirmed clinically relevant CDSS notifications were analysed to determine the reasons why they were incorrectly assessed as relevant or irrelevant by the CDSS. In Table 4, the CDSS notifications assessed as non-relevant but confirmed as relevant are shown. In Table 5, the CDSS notifications assessed as relevant but confirmed as non-relevant are shown. The actions needed to improve the sensitivity and specificity of these algorithms varies considerably and are also shown in Tables 4 and 5.

**Table 4:** CDSS notifications assessed as non-relevant but confirmed as relevant

DRP notifications	DRP strategy in handmade medication review	Reason assessed as non-relevant notification and improvement suggestion
Potassium levels—drugs inducing hyperkalemia	Stop potassium supplement with potassium level of 3.9	Cut-off point for potassium (>5.5 mmol/l) was not reached. Cut-off point needs to be refined
Benzodiazepines and fall risk	Stop or dose benzodiazepine ‘as needed’ Phase out benzodiazepine	Benzodiazepine usage should be stopped or reassessed when chronic A predictive risk algorithm for falling might be developed
Paracetamol in elderly patients in combination with risk factors	Stop paracetamol because of medication induced headaches	Include code of International Statistical Classification of Diseases into algorithm
Nortriptyline in elderly: the maximum daily dose in elderly is 50 mg. If nortriptyline is dosed higher, an ECG and monitoring of nortriptyline levels is recommended	No strategy	Two separate prescriptions of nortriptyline: 10 mg and 50 mg. The two prescriptions should be combined by the CDSS to show the total dosage
Paracetamol in elderly in combination with risk factors	Chronic use of paracetamol should be reduced to a maximum of 3 g daily	Chronic paracetamol usage in higher dosages should be avoided. Additional risk factors should be included in the algorithm alongside the dosage
Renal Failure and Amoxicillin/Clavulanic acid (oral)	Renal function 32 ml/min and oral dosage amoxicillin/clavulanic acid increased	Too low dosages when renal function improves should be included in the algorithm
Alendronic acid usage longer than 5 years	Consider whether continuation after 5 years of use is necessary	The original prescription starting date was not taken into account when patient was admitted to hospital
Citalopram in elderly	Prescribed dosage 30 mg, maximum dosage in elderly 20 mg	Two separate prescriptions citalopram; 10 and 20 mg. The two prescriptions should be combined by the CDSS in order to the total dosage
Anticoagulation therapy and INR	Increase dosage since INR is too low	The upper limit cut-off point for >5.5 INR was not reached. The algorithm focusses on toxicity, while for medication reviews a lower limit should also be included to monitor therapeutic efficacy
Potassium levels—drugs inducing hyperkalemia	Elevated potassium level of 4.6 with Losartan (which contains potassium). Converted to another ATII-antagonist	Cut-off point is set to trigger when potassium > 5.5 mmol/l. The specific prescription of losartan is not included in the algorithm of drugs containing potassium
Opioids without laxative agents. Up to 70 % of the patients using opioids experience opioid-induced constipation	Restart laxative agents when diarrhea has stopped	Prescription of laxative agents is temporarily stopped, but remains in the medication extraction. Temporarily stopped drugs should not be included in extraction. An indicator for bowel movement (stool) might be introduced

**Table 5:** CDSS notifications assessed as relevant but confirmed as non-relevant

DRP notifications	Reason scored as non-relevant	Action needed to improve algorithm
Renal failure and Rosuvastatin: contra-indicated in renal failure	Renal function was 14 ml/min with a daily dose of 10 mg Rosuvastatin, which is acceptable when the dosage is slowly increased	Introduce Rosuvastatin dosage limits of renal dysfunction into the algorithm as well as start date of prescription
Metformin and unknown vitamin B12 level	Vitamin B complex is prescribed. Vitamin B12 levels are regarded as irrelevant when supplemented	Prescription of vitamin B complex should be included in the algorithm. Furthermore, determined vitamin B12 levels should also be included in the algorithm
Tramadol and seizure: Tramadol should be used with caution in patients with a history of epilepsy and those on concomitant seizure threshold-lowering medication. Consider switching to other pain medication	Tramadol is contraindicated in epilepsy, associated drugs (nortriptyline) is prescribed for depression	Nortriptyline should be removed from the algorithm since this is not a standard therapy for epilepsy
Renal Failure and pregabalin: initial dose 75 mg per day, maximum dose 300 mg per day	Renal function of 43 ml/min with a dosage of 150 mg daily. Maximum dose was not exceeded	The algorithm should be adjusted to take into account the starting date of the prescription
Anticoagulation therapy and INR: acenocoumarol	High INR, but already given anti-dote vitamin K	Include the prescription of the anti-dote vitamin K into the algorithm
Use of acetosal, dipyridamol, clopidogrel, prasugrel without a HMG CoA-reductase inhibitor therapy (statin)	Patients were considered too old of age for HMG CoA-reductase inhibitor therapy	A frailty indicator might be considered for inclusion to determine if a HMG CoA-reductase inhibitor therapy should still be prescribed

## Discussion

This study shows that performing medication reviews in a hospitalised geriatric patient group can be of value when considering the mean of 7.0 (SD 2.2) DRP strategies identified during the gerontopharmacology meetings. The acceptance rate of the pharmacists suggestions was 74.4 % (166 out of 221 DRP suggestions) which corresponds with other described acceptance rates of DRP suggestions ranging from 39.0 to 91.6 % in long-term care patients and 65 % in hospitalised patients (Verrue et al. 2009; Mestres et al. 2015; van Dijk et al. 2009). In our study, we also investigated to which extend the DRP strategies were executed by the physician 1 week after the medication reviews. This resulted in 54.3 % (122) executed DRPs after 1 week. Another study reports that 65 % of the DRP strategies were executed (van Dijk et al. 2009). It should be mentioned that the patient group studied is easily susceptible to clinical changes, which might explain why physicians decided not to execute certain DRP strategies.

### CDSS supports medication review

Despite the absence of conclusive benefits for morbidity and mortality, medication reviews feature prominently in pharmacists’ and physicians’ daily work (Hurkens et al. 2013; de Wit et al. 2014b; Wallerstedt et al. 2014; Christensen and Lundh 2013). Many other studies have shown improvement of medication safety in elderly with the support of a CDSS (Marasinghe 2015; Ranji et al. 2014). Whether a CDSS can support the manual medication review can be shown by comparing the confirmed DRP notifications with the DRP strategies of the medication reviews. This study shows that 20 % of the DRP strategies were also identified by the CDSS. Furthermore, the CDSS identified 26 DRPs that were overlooked in the manual medication review showing that the CDSS adds value to performing the medication review manually. Meulendijk et al. (2015) also suggested that a CDSS may improve medication review effectiveness. However, the results also show the CDSS can be improved in efficiency and content.

### CDSSs strengths and weaknesses

The classification of types of medication errors shows that the notifications prompted by the CDSS, which went unrecognised during the medication reviews are strongly represented by the medication error types (c) ‘contraindications/interactions/side effects’ and error type (a) ‘indication without medication’. The importance in recognising these medication error types has also been shown by Leendertse et al. in the evaluation of the potential causes of the preventable medication-related hospital admissions. Their evaluation showed a variety of a number of potential causes like gastrointestinal problems such as bleeding and constipation and cardiovascular problems such as heart failure (Leendertse et al. 2008). These cases also involved the medication error types (a) ‘indication without medication’, and (c) ‘contraindications/interactions/side effects’, mostly missed in the medication review but recognised by the CDSS.

The medication error types (b) ‘medication without indication’, (e) ‘double medication’, and (f) ‘wrong medication’ were frequently not identified by our CDSS. Depending on the medication error type, it can be reasoned why the CDSS did not find these types of errors. The medication error type (b) requires the input of the ‘indication’. In the included medication reviews only three indications for all patients where documented in the CPOE and therefore available for the CDSS. It has already been suggested that multi-morbidity from electronic health records is poorly adopted by currents CDSSs (Fraccaro et al. 2015). The reason why the medication error types (e) ‘double medication’ and (f) ‘wrong medication’ were not found by the CDSS is because these DRPs are not included in the algorithms. These DRPs are routinely screened by the physician and pharmacist in the CPOE integrated G-standard. Our CDSS has been developed to have new notifications in addition to the CPOE notifications. The appearance of the many medication error types (e) ‘double medication’ and (f) ‘wrong medication’, suggests there is a high degree of alert fatigue with regard to CPOE alerts. Alert fatigue occurs when there are a high number of non-clinically relevant alerts, which results in the overlooking of both relevant and not-relevant alerts (van der Sijs et al. 2006). The CPOE integrated G-standard has been reported to generate 5.8 % relevant drug safety alerts (Eppenga et al. 2012). The efficiency of our CDSS is much higher with a sensitivity of 72.9 % and a specificity of 98.6 %. The analysis of why the CDSS assessed DRP notifications as non-relevant or relevant, while later evaluated as relevant and non-relevant respectively, can be used to increase the efficiency of the CDSS.

### Limitations of the study

We attempted to estimate the added value of the developed CDSS and the effectiveness of the manual medication review. Our study was limited, however, to the performed medication reviews, the CDSS-generated DRP notifications and the DRPs executed within a week after the medication review. An analysis of why DRP strategies were not executed would perhaps have provided interesting insights into the execution part of the medication review process. Furthermore, all DRPs were classified according to the type of medication error observed. A few DRPs, however, involved consultations with other physicians concerning the use of certain drugs or involved reminders to evaluate the necessity of certain drugs. We classified these DRPs as relevant, since there was considerable doubt related to the chosen therapy. This might be interpreted differently in other studies.

### Further research

Using automation to make medication reviews more efficient is considered highly necessary according to a recent survey (de Wit et al. 2014b). There remain several differences between CDSSs both content and efficiency of current CDSS varies considerably. Some CDSSs still rely on the manual input of a single patient’s details such as medical history, medication and pathology. These CDSSs do support the medication review but are lacking in terms of time efficiency (de Wit et al. 2014a). The effectiveness of our CDSS to support medication reviews needs to be increased by 1) complementing the content with overlooked DRP strategies from the medication review and 2) by optimising the DRP notifications that were incorrectly assessed by the CDSS.

Furthermore, the results of this study show that certain DRP notifications are correct when strictly following the applicable guideline, but are found to be irrelevant after discussion in the gerontopharmacology meeting. The development of algorithms allowing discriminating between patients to initiate ‘deprescribing’ or to specifically not initiate pharmacotherapy treatment will be a challenge. Deprescribing aims to reduce the use of drugs that are less beneficial, or even, detrimental taking into account the individual needs for therapy (Scott et al. 2015). The challenge of incorporating ‘deprescribing’ is shown by the lack of DRP notifications in medication error type (b) ‘medication without indication’. An example of deprescribing and of not initiating pharmacotherapy treatment is the prescription of statins. In our study, treatment with HMG CoA-reductase inhibitor (statins) was not initiated in ten patients because they were too frail and too old. The benefit of stopping statins or not initiating statins when life expectancy is limited should be considered in CDSSs (Holmes and Todd 2015). The development of algorithms approaching the level of expertise in the gerontopharmacology meeting will be a great challenge and improvement for the CDSS.

## Conclusions

Performing medication reviews in a hospitalised geriatric patient group can be of value seen the high number of accepted DRP strategies. More than half of the accepted DRP strategies were executed within 1 week. The distribution of the relevant medication error types shows the strengths and weaknesses of the CDSS compared to the manual medication review in this study. Our developed CDSS is currently unable to replace the manual medication review. It can however be of additional value for the manual medication review. Further development of the current CDSS is needed to fully support manual medication reviews.

## Abbreviations

CDSS: clinical decision support system
DRPs: drug-related problems
ADR: adverse drug reactions
CPOE: computerised physician order entry
IGZ: Dutch Healthcare Inspectorate
KNMP: Royal Dutch Association for the Advancement of Pharmacy
